# Protocol for the identification and expression analysis of a cytoplasmic membrane-localized protein STING

**DOI:** 10.1016/j.xpro.2023.102172

**Published:** 2023-03-20

**Authors:** Jiaqi Shi, Caiqi Liu, Shengnan Luo, Yingying Zhang, Mingwei Li, Meng Zhou, Mingjiao Weng, Xiaobo Li, Tongsen Zheng

**Affiliations:** 1 Department of Phase 1 Trials Center, Harbin Medical University Cancer Hospital, No.150 Haping Road, Nangang District, Harbin, Heilongjiang 150081, China; 2 Key Laboratory of Molecular Oncology of Heilongjiang Province, No.150 Haping Road, Nangang District, Harbin, Heilongjiang 150081, China; 3 Department of Gastrointestinal Medical Oncology, Harbin Medical University Cancer Hospital, No.150 Haping Road, Nangang District, Harbin, Heilongjiang 150081, China; 4 Department of Pathology, Harbin Medical University, No.157 Baojian Road, Nangang District, Harbin, Heilongjiang 150081, China

**Keywords:** Cell Biology, Cell isolation, Flow Cytometry/Mass Cytometry, Cell Membrane, Cancer, Sequencing, Immunology, Microscopy, Molecular Biology, Protein Biochemistry

## Abstract

Here, we present a protocol for the detection of the two STING isoforms (erSTING and pmSTING) in human peripheral blood mononuclear cells or mouse splenocytes using Western blot and PCR. We detail steps to construct plasmids encoding each isoform and transfer them into mouse and human cell lines. Finally, we describe how to detect cell membrane localization of pmSTING using flow cytometry, immunoprecipitation, and immunofluorescence. This protocol is applicable for proteins with well-predicted topological structures.

For complete details on the use and execution of this protocol, please refer to Li et al.^1^.

## Highlights

• Protocol for identification, construction, and expression analysis of new isoform
• Simple, rapid, direct detection of cytomembrane-localized proteins
• Applicable to probing a novel STING isoform (pmSTING) in the plasma membrane

## Abstract (editor highlights)

Publisher’s note: Undertaking any experimental protocol requires adherence to local institutional guidelines for laboratory safety and ethics.

## Graphical abstract

**Figure:**
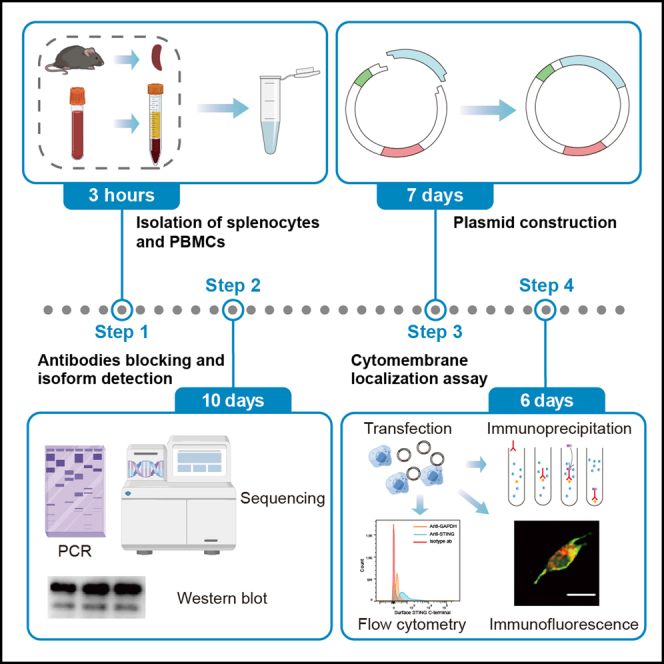

## Before you begin

### Institutional permissions

The protocol requires spleen tissue derived from C57BL/6J wild type (WT) mice and STING-deficient mice (C57BL/6J-*Sting1*^*gt*^/J) and blood from healthy volunteers. Ethical approvals are required before starting this procedure. All animal experiments were carried out with the approval of the Harbin Medical University Research Ethics Committee.

### Cell culture

**Timing: 30 min**

1. Prepare buffers. DMEM (Gibco) supplemented with 10% fetal bovine serum (FBS) (Gibco) and 1% Penicillin-Streptomycin solution (Biosharp). RPMI 1640 (Gibco) added with 10% FBS and 1% Penicillin-Streptomycin solution.
2. Standard cell culture conditions (37°C, 5% CO2).
3. HEK293T cell line and B16-Blue™ ISG-KO-STING cell line (Invivogen).

### Animal models

**Timing: 14 days**

4. C57BL/6J WT mouse was purchased from the second affiliated hospital of Harbin Medical University.
5. C57BL/6J-*Sting1*^*gt*^/J mouse is an I199N missense mutant allele of the Sting gene, which was purchased from Jackson Laboratory.
6. C57BL/6J WT mouse and STING-deficient mouse were raised in a specific pathogen free (SPF) environment to isolate splenocytes.

## Key resources table

**Table:** 

REAGENT or RESOURCE	SOURCE	IDENTIFIER
**Antibodies**		

Rat monoclonal FITC anti-mouse CD3 (clone 17A2) (ICC 1:50)	BioLegend	Cat#100204; RRID: AB_312661
Rat monoclonal FITC anti-mouse CD19 (clone 6D5) (ICC 1:50)	BioLegend	Cat#115506; RRID: AB_313641
Rat monoclonal FITC anti-mouse CD11b (clone M1/70) (ICC 1:200)	BioLegend	Cat#101206; RRID: AB_312789
Rabbit polyclonal anti-STING for C-terminal domain of erSTING and pmSTING in human and mouse (WB 1:600; F: 2 μg/mL/10^7^ cells; IP: 5 μg/mL)	Proteintech	Cat#19851-1-AP; RRID: AB_10665370
Rabbit polyclonal anti-STING for C-terminal domain of erSTING and pmSTING in human and mouse (F: 2 μg/mL/10^7^ cells; IP: 5 μg/mL)	Abcam	Cat#ab92605; RRID: AB_10562137
Rabbit polyclonal anti-STING for C-terminal domain of erSTING and pmSTING in human and mouse (F: 2 μg/mL/10^7^ cells; IP: 5 μg/mL)	Novus	Cat#NBP2-24683; RRID: AB_2868483
Rabbit polyclonal anti-STING for C-terminal domain of erSTING and pmSTING in human and mouse (ICC 1:200)	Abcam	Cat#ab189430
Rabbit polyclonal IgG isotype (F: 2 μg/mL/10^7^ cells; IP: 5 μg/mL)	Sigma-Aldrich	Cat#12-370; RRID: AB_145841
Rabbit monoclonal anti-Flag (ICC 1:400)	Cell Signaling Technology	Cat#14793S; RRID: AB_2572291
Mouse monoclonal anti-GAPDH (F: 2 μg/mL/10^7^ cells; IP: 5 μg/mL)	Proteintech	Cat#60004-1-Ig; RRID: AB_2107436
Goat polyclonal anti-mouse IgG, HRP-conjugate (WB 1:2000; IP 1:2000)	Proteintech	Cat#SA00001-1; RRID: AB_2722565
Goat polyclonal anti-rabbit IgG, HRP-conjugate (WB 1:2000; IP 1:2000)	Proteintech	Cat#SA00001-2; RRID: AB_2722564
Goat polyclonal anti-rabbit IgG, Alexa Fluo 594-conjugate (ICC 1:200)	Abcam	Cat#ab150080; RRID: AB_2650602

**Biological samples**		

PBMCs	This paper, donors	N/A

**Chemicals, peptides, and recombinant proteins**		

WGA lectin (FITC)	GeneTex	Cat#GTX01502
DMEM high glucose medium	Gibco	Cat#11995065
Fetus bovine serum (FBS)	Gibco	Cat#10091148
Penicillin-Streptomycin solution	Biosharp	Cat#P917928
RPMI 1640	Gibco	Cat#11875093
PBS	Solarbio	Cat#P1020
RBC lysis buffer	TBD	Cat#NH4CL2009
Ficoll Paque Plus	Cytiva	Cat#17144003
RIPA buffer	Thermo Fisher	Cat#89901
PMSF	Roche	Cat#11697498001
Phosphatase inhibitors	Roche	Cat#4906845001
PVDF membrane	Millipore	Cat#IPVH00010
BSA	BioFroxx	Cat#4240
jetPRIME transfection regent	Polyplus Transfection	Cat#PT-114-07
Trizol reagent	Invitrogen	Cat#15596018
Reverse Transcriptase M-MLV (RNase H)	TAKARA	Cat#H2640A
4% paraformaldehyde (PFA)	Biosharp	Cat#BL539A
DAPI	Solarbio	Cat#C0065
Tris	Biotopped	Cat#T6061
SDS	Biosharp	Cat#BS088
Glycine	Biosharp	Cat#BS082
75% ethanol	Aladdin	Cas#64-17-5
Tween-20	Biosharp	Cas#9005-64-5
SlowFade™ Gold antifade reagent	Thermo Fisher Scientific	Cat#S36940

**Critical commercial assays**		

PAGE Gel Fast Preparation Kit	Epizyme	Cat#PG112
2×Pfu Mix	Sciencestar	N/A
ECL kit (femtogram)	Affinity	Cat#KF003
BCA Kit	Beyotime Institute of Biotechnology	Cat#P0010

**Experimental models: Cell lines**		

Human: HEK293T	Cell Culture Center of Chinese Academy of Medical Science	Cat#SCSP-502
Mouse: B16-Blue™ ISG-KO-STING	Invivogen	Cat#bb-kostg

**Experimental models: Organisms/strains**		

Mouse: C57BL/6J WT (Female mouse aged 6 weeks)	The Animal Center of the Second Affiliated Hospital of Harbin Medical University	N/A
Mouse: C57BL/6J-*Sting1*^*gt*^/J (Female mouse aged 6 weeks)	Jackson Laboratory	RRID: IMSR_JAX:017537

**Oligonucleotides**		

Primer: Mouse Tmem173 Forward: GCTGTGCCATGTCCAGTC	This paper, Genewiz	N/A
Primer: Mouse Tmem173 Reverse: CAACCGCAAGTACCCAAT	This paper, Genewiz	N/A
Primer: Human Tmem173 Forward: TCTCCTCGTCATCATCCAG	This paper, Genewiz	N/A
Primer: Human Tmem173 Reverse: AGGAGGATGTTCAGTGCC	This paper, Genewiz	N/A

**Recombinant DNA**		

Plasmid: human erSTING-Flag	This paper, Genewiz	N/A
Plasmid: human erSTING-EGFP	This paper, Genewiz	N/A
Plasmid: human pmSTING-Flag	This paper, Genewiz	N/A
Plasmid: human pmSTING-EGFP	This paper, Genewiz	N/A
Plasmid: mouse erSTING-Flag	This paper, Genewiz	N/A
Plasmid: mouse erSTING-EGFP	This paper, Genewiz	N/A
Plasmid: mouse pmSTING-Flag	This paper, Genewiz	N/A
Plasmid: mouse pmSTING-EGFP	This paper, Genewiz	N/A

**Software and algorithms**		

FlowJo 10.6.1	BD	https://www.flowjo.com/solutions/flowjo/downloads
Bio-Rad Gel Doc XR + system	Bio-Rad	https://www.bio-rad.com/zh-cn/product/gel-doc-xr-gel-documentation-system?ID=O494WJE8Z
Image Lab Software 6.1	Bio-Rad	https://www.bio-rad.com/zh-cn/category/image-lab-software-resources?ID=PJWA0VTU86LJ

**Other**		

70 μm cell strainer	Corning	Cat#431751
100 μm cell strainer	Corning	Cat#431752
EDTA anti-coagulation tubes	BD	Cat#367863
6-well cell culture plates	Corning	Cat#3506
Axygen® 1.5 mL MaxyClear SnapLock	Corning	Cat#MCT-150-C-S
15 mL conical centrifuge tubes	Corning	Cat#SCT-15ML-R-S
Single-use 5 mL polypropylene syringe	Sigma-Aldrich	Cat#Z116866
Polylysine-treated slides	Sigma-Aldrich	Cat#P0425
Hydrophobic barrier pen	Yeasen	Cat#36310ES64
5 mL Pasteur pipettes	Sorfa	Cat#320511
Nikon C2 confocal microscope	Nikon	https://www.microscope.healthcare.nikon.com/zh_CN/products/confocal-microscopes/c2
BD LSR Fortessa	BD	https://www.bdbiosciences.com/zh-cn/products/instruments/flow-cytometers/research-cell-analyzers/bd-lsrfortessa

## Materials and equipment

### Buffers

**Table:** DMEM culture medium

Reagent	Final concentration	Amount
DMEM	89%	89 mL
fetus bovine serum	10%	10 mL
Penicillin-Streptomycin	1%	1 mL
**Total**		**100 mL**

The culture medium can be stored at 4°C for 1 month.

**Table:** RPMI 1640 culture medium

Reagent	Final concentration	Amount
RPMI 1640	89%	89 mL
fetus bovine serum	10%	10 mL
Penicillin-Streptomycin	1%	1 mL
**Total**		**100 mL**

The culture medium can be stored at 4°C for 1 month.

### 5% BSA

5% BSA solution: add 5 g BSA in 100 mL ddH_2_O. Store at −18°C to −20°C for 1 month.

### 1% BSA

1% BSA solution: add 1 g BSA in 100 mL ddH_2_O. Store at −18°C to −20°C for 1 month.

### PBST

PBS supplemented with Tween-20: PBS with 0.1% of Tween-20. Store at 20°C for 1 month.

### 2% paraformaldehyde (PFA)

2% PFA solution: add 100 mL 4% PFA in 100 mL PBS. Store at 20°C for 3 months.

## Step-by-step method details

### Isolation of human peripheral blood mononuclear cells (PBMCs)

**Timing: 1–2 h**

Here, we describe how to isolate PBMCs from the peripheral blood of healthy volunteers.

**Figure 1:**
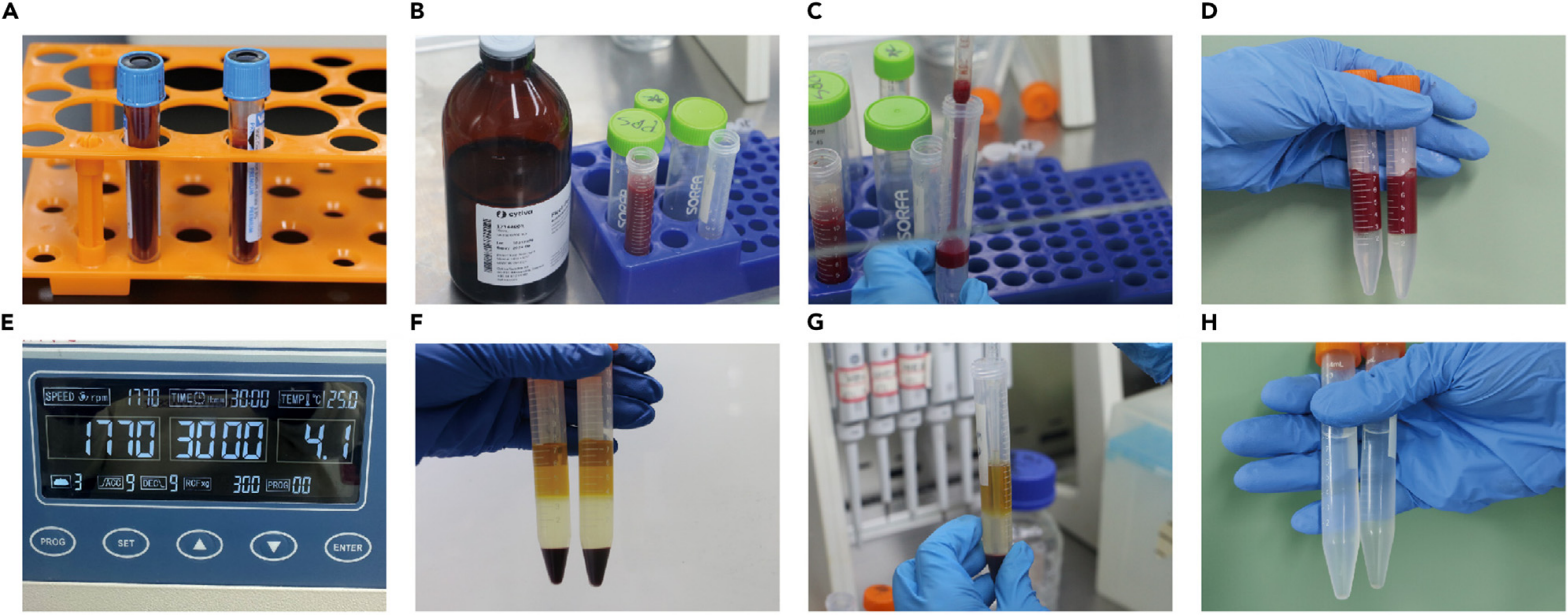
Isolation of human peripheral blood mononuclear cells (PBMCs) (A) Peripheral blood was collected from healthy donors. (B) Peripheral blood was diluted 1:1 with PBS. (C) Carefully layer the diluted blood sample (4 mL) on Ficoll-Paque solution. (D) Do not mix Ficoll-Paque with the diluted blood sample. (E) Centrifuge at 300 g for 30 min at 25°C. (F) After centrifugation, the tube is divided into three layers. The upper layer is plasma and PBS, the lower layer is mainly red blood cells and granulocytes, and the middle layer is Ficoll-Paque solution. There is a narrow band of white cloud layer dominated by mononuclear cells (PBMCs) at the interface between the upper and middle layers. (G) Slow and gentle absorption of PBMCs layer with a pasteur pipette. (H) Resuspended in PBS and centrifuged to collect PBMCs.

1. Collect 3 mL blood into 5 mL EDTA anti-coagulation tubes and dilute with sterile PBS to 6 mL in a clean tube.
2. Then gently add the diluted 4 mL blood in the tube containing 4 mL Ficoll-Hypaque solution on the bottom.
3. Centrifuged the tube at 300 *g*, 30 min (troubleshooting 1).
4. After centrifugation, divide the solution in the tube into three layers, transfer the intermediate foggy layer to a blank tube and undergo another centrifugation at 300 *g*, 10 min.
5. Remove the RBCs by resuspending the cell precipitation in 1 mL RBC lysis buffer for 2 min.
6. Resuspend the cell precipitation with 5 mL PBS after centrifugation at 300 *g* (Figure 1).

**CRITICAL:** These steps must be performed under sterile conditions.

### Isolation of splenocytes

**Timing: 1–2 h**

This part details how to isolate splenocytes from the spleens of C57BL/6J WT mice and C57BL/6J-*Sting1*^*gt*^/J mice.

**Figure 2:**
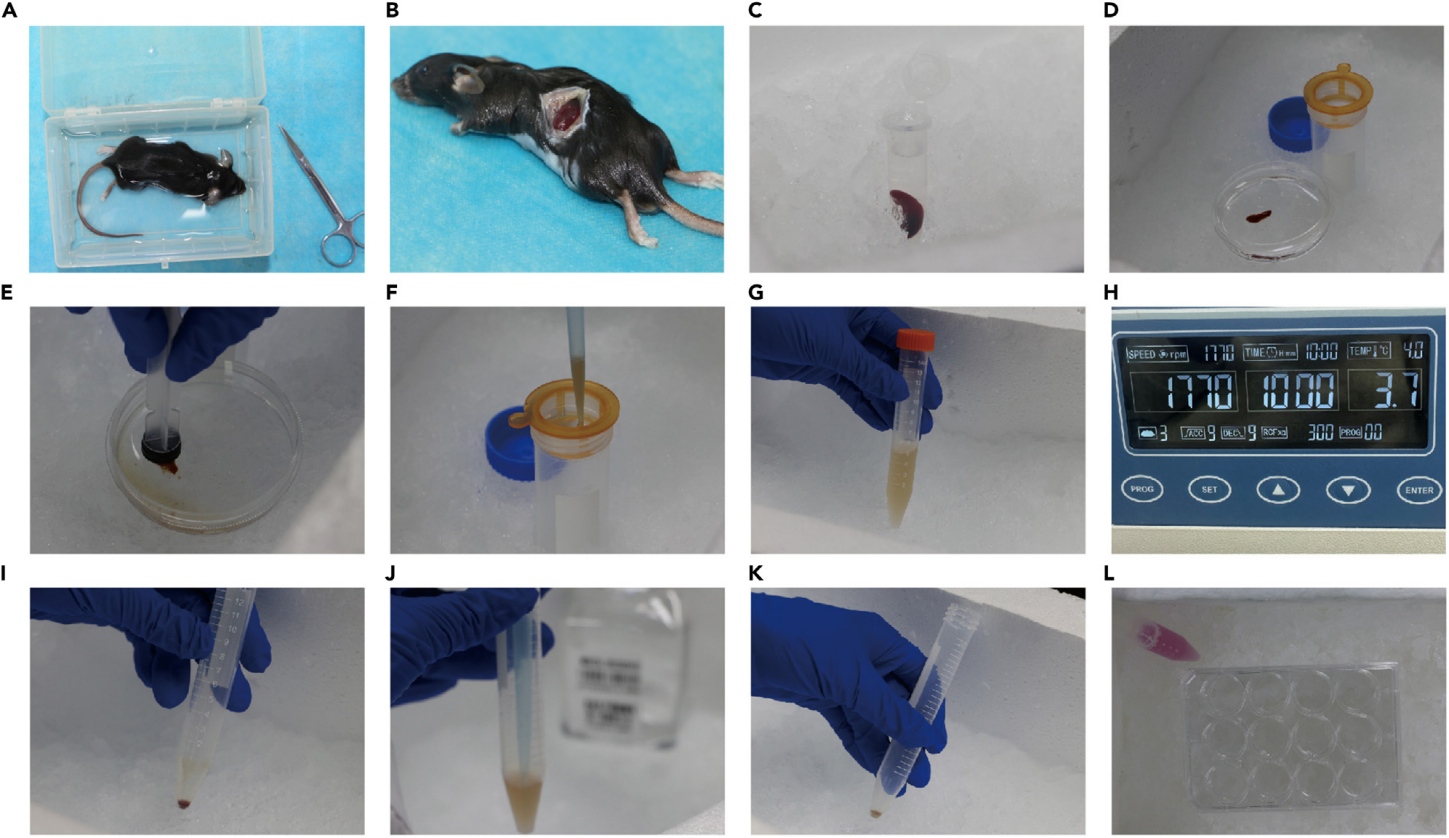
Isolation of mouse splenocytes (A) Mouse was sacrificed and immersed in 75% ethanol. (B) Exposure of the spleen. (C) Spleen in a sterile collection tube. (D) Place the spleen in a sterile dish containing 4 mL PBS. (E) Grind the spleen. (F) Transfer the ground spleen cells to a cell strainer. (G) Single-cell suspension of spleen cells. (H) Centrifuge at 300 g for 10 min at 4°C. (I) Discard the supernatant. (J) Add 2 mL RBC lysis buffer. (K) Centrifuge and discard the supernatant. (L) Resuspension and counting of splenocytes.

7. Sacrifice and immerse the mouse in 75% ethanol, then expose the spleen.
8. Harvest fresh spleens from WT or C57BL/6J-*Sting1*^*gt*^/J mice and gently crush them in sterile PBS by the inner piston of the syringe.
9. Then suspend splenocytes in 10 mL PBS, filtrate them with the filter mesh (100 μm), and concentrate at 300 g, 10 min.
10. Remove the RBCs by resuspending cell precipitation in 1 mL RBC lysis buffer for 2 min.
11. After concentrating twice at 300 g, resuspended the cells with 5 mL PBS.
12. Resuscitate splenocytes by 4 mL RPMI 1640 and count for follow-up experiments (troubleshooting 2) (Figure 2).

**CRITICAL:** After the mice were killed, the spleens were removed after disinfection with alcohol and operated under aseptic conditions.

### Western blot (WB)

**Timing: 2 days**

This part details how to detect different STING isoforms in human PBMCs or C57BL/6 mouse splenocytes by WB.

13. Extract total proteins using RIPA buffer added with protease inhibitor PMSF and phosphatase inhibitors, and the final concentration of PMSF in protein solution is 0.2 mM. Add 100 μL precooled RIPA lysate per million cells.
14. Use a BCA Kit to quantify the proteins.
15. Separate equal amount of protein by electrophoresis and transfer onto a PVDF membrane, 50 μg protein loading for PBMCs and 40 μg protein loading for splenocytes.
16. Block membranes with 5% BSA at 25°C for 1 h. Incubated with anti-STING rabbit polyclonal antibody (19851-1-AP) diluted at 1: 600 in PBST and incubated for 14 h at 4°C.

***Note:*** PVDF membranes can also be incubated with anti-STING rabbit polyclonal antibody (19851-1-AP) diluted at 1: 600 in PBST for 2 h at 25°C.

17. Wash the membranes three times by PBST 10 min each time, and incubate them with peroxidase-conjugated secondary antibodies (SA00001-1 and SA00001-2, diluted at 1: 2000 in PBST) for 1 h at 37°C.

***Note:*** Increasing the number of washing times properly can decrease the background.

18. Wash the membranes three times by PBST for 10 min each time. Image with the ECL kit (KF003) and Bio-Rad Gel Doc XR + system.

**Pause point:** The proteins can be stored at −80°C for 6 months.

### PCR

**Timing: 2 h**

This section details the detection of 2 distinct N-terminal STING isoforms in human PBMCs or C57BL/6 mouse splenocytes by PCR.

19. Extract total RNA by Trizol reagent according to the manufacturer’s instructions (https://assets.thermofisher.cn/TFS-Assets/LSG/manuals/trizol_reagent.pdf), more than one million cells per sample.

**Pause point:** Resulting RNA can be stored at −80°C for 1 month.

20. Use 1 μg of total RNA to synthesize cDNA using Reverse Transcriptase M-MLV (RNase H) following the manufacturer’s protocol (https://www.takarabiomed.com.cn/DownLoad/2641A.pdf).

**Pause point:** Resulting cDNA can be stored at −20°C for 1 year.

21. Set up the PCR reaction as follows.

**Table:** PCR reaction mixture

Reagent	Final concentration	Amount
cDNA	5%	1 μL
2×Pfu Mix	50%	10 μL
Primers	10%	2 μL
nuclease-free water	35%	7 μL
**Total**		**20 μL**

For the PCR reaction mixture, please make a temporary configuration when needed.

***Note:*** Primers were included in the Key resource table.

22. Run the PCR.

**Table:** PCR cycling conditions

Steps	Temperature	Time	Cycles
Initial Denaturation	94°C	30 s	1
Denaturation	94°C	10 s	35 cycles
Annealing	60°C	30 s	
Extension	72°C	1 min	
Final extension	72°C	5 min	1
Hold	4°C	indefinite

23. Run PCR products on gel electrophoresis. Extract the corresponding positions of erSTING and pmSTING.

***Note:*** NCBI database, Human erSTING 2170 bp, Human pmSTING 1943 bp; mouse erSTING 2302 bp, mouse pmSTING 2179 bp.

24. Send the gel bands of erSTING and pmSTING to the Genewiz company for sequencing.
25. Compare the sequencing results with the sequences in the NCBI database.

### Flow cytometry

**Timing: 2 days**

This section details how to use flow cytometry to detect the cell surface STING.

26. To identify the plasma membrane STING isoform, incubate human PBMCs and mouse splenocytes (one million cells per group) with 0.2 μg anti-STING antibody (19851-1-AP; ab92605; NBP2-24683), anti-GAPDH antibody (60004-1-Ig) and rabbit IgG isotype (12–370) per group diluted by PBS at 4°C for 1 h (troubleshooting 3).
27. After washing twice with 1 mL PBS, stain the cells with the FITC-conjugated secondary antibody (SA00003-2, 1:200) diluted with PBS at 25°C in a dark place for 1 h.
28. After staining, wash the cells twice with PBS (300 g, 5 min) and resuspend them with 500 μL PBS, then filter the cells with the filter mesh (50 μm).
29. Examine all samples by BD LSR Fortessa and analyze them with FlowJo 10.6.1 software (TreeStar, Inc.).

**CRITICAL:** Please gently operate and don't destroy the cells.

### Cytomembrane protein co-staining

**Timing: 2 days**

This section details how to detect the cytomembrane co-localization of STING isoform and classical membrane protein by immunofluorescent staining.

30. The C57BL/6J WT and C57BL/6J-*Sting1*^*gt*^/J mice splenocytes smear samples preparation.
  a. Prepare clean polylysine-treated slides, blow the ten million cells evenly with 1 mL PBS, absorb the 30 μL suspension liquid with a pipette, drop it on the slide, let the liquid spread freely, form a monolayer cell structure, and let the cell suspension dry freely at 25°C.
  b. Use a hydrophobic barrier pen to circle the edge of the dry liquid surface to avoid fluid loss during subsequent operations.
  c. Use 2% cold paraformaldehyde (PFA) to fix the slides for 5 min and then use 1% BSA to block the slides in PBS for 30 min.
31. Wash the slides with cold PBS twice, and ensure the cells on the slides are submerged in PBS. Perform the staining with anti-STING antibody (ab189430, 1:200) in PBS for 14 h at 4°C and fluorochrome-conjugated antibodies against cell surface markers at 4°C in a dark place for 30 min: FITC anti-mouse CD3 antibody (100204, 1:50), FITC anti-mouse CD19 antibody (115506, 1:50), FITC anti-mouse CD11b antibody (101206, 1:200).
32. Wash the slides with cold PBST three times, and ensure the cells on the slides are submerged in PBST. Stain the samples with Alexa Fluo 594-conjugated goat anti-rabbit IgG (ab150080, 1:200) at 25°C in a dark place for 1 h.
33. Perform the nuclear staining by DAPI at 25°C for 10 min.

***Note:*** Adjust the staining time of DAPI to avoid overstaining.

34. Finally, wash the samples with cold PBST, make sure the cells on the slides are submerged in PBST, and then seal them with a sealing agent.

***Note:*** Gently wash the slides to retain more cells.

35. Capture images using Nikon C2 confocal microscope.

### Plasmid construction

**Timing: 7 days**

This part details the method for erSTING and pmSTING plasmid construction.

36. Synthesize de novo the DNA sequence encoding mouse or human erSTING ORF fused EGFP or 3×Flag, and mouse or human pmSTING ORF fused EGFP or 3×Flag respectively, and then clone them into pcDNA3.1 vector.
37. Transfect the human erSTING-EGFP, erSTING-3×Flag or pmSTING-EGFP, pmSTING-3×Flag plasmid into HEK293T cells by using jetPRIME transfection regent according to manufacturer’s instruction (https://www.polyplus-transfection.com/wp-content/uploads/2015/08/Polyplus-Short-Protocol-jetPRIME-DNA.pdf) to express erSTING or pmSTING, independently.
38. Transfect the mouse erSTING-EGFP, erSTING-3×Flag or pmSTING-EGFP, pmSTING-3×Flag plasmid into B16-Blue™ ISG-KO-STING cells (Invivogen) to express erSTING or pmSTING, independently. 200 000 cells in 6-well plates per well, transfect cells at 60% confluency, 2 μg plasmid DNA per well.

### Immunofluorescence

**Timing: 2 days**

This section details how to detect the cytomembrane localization of STING isoform by immunofluorescent staining.

39. 24 h after transfection, fix erSTING-Flag, pmSTING-Flag or Flag plasmid transfected B16-Blue™ ISG-KO-STING or HEK293T cells by 2% cool PFA for 5 min and block them by 1% BSA for 30 min.
40. Wash the samples with cold PBS twice, make sure the cells on the slides are submerged in PBS, and incubate the slides by primary antibody against Flag (14793S, 1:400) for 14 h at 4°C.
41. Rewarm at 25°C for 20 min. Then stain the slides with WGA Lectin (FITC) (GTX01502, 1:500) diluted with PBS for 15 min at 25°C in a dark place.

***Note:*** Wheat germ agglutinin (WGA) is usually used to label glycoproteins. It can be coupled with N-acetyl-β-D-glucosamine residues and N-acetyl-β-D-glucosamine oligomers on the cytomembrane. It is usually used to label the cytomembrane of mammalian cells.^2^

42. Wash the slides with cold PBST three times, make sure the cells on the slides are submerged in PBST, and stain the slides with Alexa Fluo 594-conjugated goat anti-rabbit IgG (ab150080, 1:200) diluted with PBS at 25°C in a dark place for 1 h (troubleshooting 4).
43. Perform the nuclear staining by DAPI at 25°C for 10 min.
44. Finally, wash the slides with cold PBST and seal them with a sealing agent.
45. Capture images using Nikon C2 confocal microscope.

### Immunoprecipitation (IP)

**Timing: 2 days**

This part details how to perform immunoprecipitation to detect STING isoform expressed on the cytomembrane of human PBMCs or mouse splenocytes.

46. Transfect pmSTING-EGFP or erSTING-EGFP into B16-Blue™ ISG-KO-STING cells using jetPRIME transfection regent according to manufacturer’s instruction (https://www.polyplus-transfection.com/wp-content/uploads/2015/08/Polyplus-Short-Protocol-jetPRIME-DNA.pdf).

200 000 cells in 6-well plates per well, transfect cells at 60% confluency, 2 μg plasmid DNA per well.

47. 24 h after transfection, incubate the cells with 5 μg/mL rabbit anti-STING antibody (19851-1-AP), rabbit anti-GAPDH antibody (60004-1-Ig), rabbit IgG isotype (12–370) in PBS at 4°C for 1 h.
48. Collect the cell pellets by centrifugation at 6000 g for 5 min and then split them with RIPA buffer containing PMSF on ice for 10 min.
49. Centrifugate at 4°C, 10,000 g, for 20 min to collect the supernatant.
50. Use the HRP-conjugated goat anti-rabbit IgG (SA00001-2, 1:2000) to detect the IgG binding on the cell membrane (troubleshooting 5).

## Expected outcomes

You can obtain information about mRNA and protein sequences of both human and mouse erSTING^3^^,^^4^ and pmSTING from the NCBI websites and their topological schematic from our previously published paper.^1^ The STING isoforms detection results by WB and PCR are shown in our paper.^1^ It clearly shows that there are two STING isoforms with different molecular weights in both mouse splenocytes and human PBMCs. The WB results show that the molecular weight of both mouse STING isoforms is 37 kD (erSTING) and 34 kD (pmSTING), respectively (Figure 3B in ref.^1^), whereas the molecular weight of both human STING isoforms is 39 kD (erSTING) and 34 kD (pmSTING) respectively (Figure 5F in ref.^1^). The PCR results show that the amplified fragment length of both mouse STING isoforms is 301 bp (erSTING) and 178 bp (pmSTING), respectively (Figure 3C in ref.^1^), whereas the amplified fragment length of both human STING isoforms is 446 bp (erSTING) and 219 bp (pmSTING) respectively (Figure 5G in ref.^1^).

The results of pmSTING detection with flow cytometry are shown in our paper.^1^ Suppose a cytomembrane-localized STING isoform (pmSTING) exists in mouse splenocytes or human PBMCs. Flow cytometry results will show a shift of the staining of mouse pmSTING (Figure 2A in ref.^1^) or human pmSTING (Figure 5A in ref.^1^) by using an antibody against the C-terminal epitope of STING compared to the control staining by using isotype or anti-GAPDH antibodies.

The immunofluorescence results of co-staining of pmSTING with cytomembrane protein markers of different immune cells in C57BL/6J WT or C57BL/6J-*Sting1*^*gt*^/J mouse splenocytes are shown in our paper.^1^ You can observe a strong co-localization between pmSTING and the cytomembrane protein markers (such as CD3, CD19 and CD11b) in non-permeabilized C57BL/6J WT splenocytes (Figure 2C in ref.^1^) in contrast, and you will fail to observe the co-localization between them in splenocytes of the C57BL/6J-*Sting1*^*gt*^/J mouse (Figure 2D in ref.^1^).

After transfection of Flag, erSTING-Flag or pmSTING-Flag into B16-Blue™ ISG-KO-STING or HEK293T cells, use the anti-Flag antibody to detect Flag projecting outside cells by immunofluorescence. The staining results show that only the cells transfected with human pmSTING-Flag are red fluorescence staining positive on the non-permeabilized HEK293T cells membrane, and it co-localizes with the cytomembrane staining marker WGA (Figure 3F in ref.^1^). The staining results are the same in the mouse B16-Blue™ ISG-KO-STING cells transfected with mouse pmSTING-Flag (Figure 5H in ref.^1^).

The immunoprecipitation results are also shown in our paper.^1^ If there is a cytomembrane-localized isoform (pmSTING), the WB results will show the presence of IgG immunoblotting band only in the STING antibody immunoprecipitated lysates by using an anti-IgG antibody (Figure 3E in ref.^1^), after immunoprecipitation with the specific antibodies against STING C-terminal domain, against isotype or GAPDH.

## Quantification and statistical analysis

For relevant analysis of cytomembrane-localized proteins, we recommend analyzing and presenting them in several different ways, such as immunofluorescence, co-localization, flow cytometry and immunoprecipitation, to ensure the accuracy of pmSTING localization and avoid the non-specific results of one experimental method.

For flow cytometry and immunofluorescence analysis, ensure that the number of cells, antibody dosage and concentration in each group were consistent.

## Limitations

This method is unsuitable if the membrane protein is evenly spanned and the N or C terminus of the protein is located in the cytoplasm rather than toward extracellular,^5^ and if it is difficult to obtain specific antibodies against the extracellular segment.

The spatial structure of the protein may mask specific epitopes, which may result in the use of a particular IP antibody, and the target protein complex will rarely be precipitated, no matter how much the antibody concentration is increased. Since the test is performed in the natural state, the proteins pulled down by IP may be different at different times and under different treatments, and of course, the accuracy of the protein obtained will become more and more significant as the number of experiments increases.

## Troubleshooting

### Problem 1

Ficoll separation stratification confusion, Low amount of extracted PBMCs.

### Potential solution

The diluted blood needs to be added gently to the upper layer of the ficoll in the centrifuge tube, it must be done gently to avoid mixing the two solutions, and eventually, it must be ensured that the two solutions are clearly layered. When separating PBMCs in the first step of centrifugation, note that the descending speed setting must be set to NO BREAK, or only 1–2% brake. Otherwise, the stratification will be confused.

### Problem 2

The number of surviving splenocytes is too small.

### Potential solution

During the grinding process, try to control the grinding force to ensure that the cell sieve is suspended and avoid mass cell death caused by direct grinding at the bottom of the dish. To ensure the vitality of cells, the whole operation process should be gentle to avoid causing mechanical damage to cells. To keep the cells in good condition, the whole experimental cycle should be shortened as much as possible.

### Problem 3

The signals of cell surface markers are undetectable or inadequate.

### Potential solution

Set up appropriate controls during flow cytometry analysis (refer to step-by-step method details 26): negative control to avoid false positive (anti-GAPDH), isotype control to eliminate background staining caused by non-specific binding of antibodies to the cell surface, positive control (if needed). In addition, be sure to add the secondary antibody at the recommended concentration and target the correct host of the primary antibody. If the membrane protein expression level is low, choose a bright fluorescent secondary antibody (such as PE) to detect it.

### Problem 4

The stain was too strong or too weak during the immunofluorescence.

### Potential solution

Following the experimental conditions of the antibody instructions, the antigen should be fully repaired and the epitope exposed to avoid false negative results. Select the appropriate antibody concentration, thoroughly wash off the excess antibodies, and avoid the broad background of the fluorescence image being too high.

### Problem 5

More protein impurity bands precipitation.

### Potential solution

Increase the concentration of salt ions in the washing buffer, appropriately increase the elution times, and reduce the incubation time. Pay attention to the molecular weight of the target protein.

## Resource availability

### Lead contact

Further information and requests for resources and reagents should be directed to and will be fulfilled by the lead contact, Tongsen Zheng (zhengtongsen@hrbmu.edu.cn).

### Materials availability

This study did not generate new unique reagents.

### Data and code availability

This study did not generate/analyze datasets/code.
